# Identification of immune-related endoplasmic reticulum stress genes in proliferative diabetic retinopathy using bioinformatics analysis

**DOI:** 10.3389/fendo.2024.1341206

**Published:** 2024-08-30

**Authors:** Han Chen, Enguang Chen, Miaomiao Liu, Jianhui Wang, Jiawei Yin, Peiquan Zhao, Yu Xu

**Affiliations:** Department of Ophthalmology, Xinhua Hospital Affiliated to Shanghai Jiao Tong University School of Medicine, Shanghai, China

**Keywords:** proliferative diabetic retinopathy, endoplasmic reticulum stress, biomarkers, differentially expressed genes, bioinformatics, drug prediction

## Abstract

**Background:**

Proliferative diabetic retinopathy (PDR) is a severe complication of diabetes, and understanding its molecular mechanisms is crucial. Endoplasmic reticulum (ER) stress has been implicated in various diseases, including diabetic complications. This study aims to elucidate ER stress-related biomarkers in PDR, providing insights into the underlying molecular pathways.

**Methods:**

We analyzed two independent PDR datasets, GSE102485 and GSE60436. The GSE102485 dataset (22 PDR and 3 normal samples) was the primary dataset for comprehensive analyses, including differential expression, functional enrichment, PPI network construction, immune cell infiltration, and drug prediction. The GSE60436 dataset (6 PDR and 3 normal samples) was used for validation. *In vitro* experiments using human umbilical vein endothelial cells (HUVECs) in a high-glucose environment were conducted to validate key bioinformatics outcomes. Western blotting assessed protein levels of ER stress markers (TRAM1 and TXNIP).

**Results:**

Differential expression analysis identified 2451 genes, including 328 ER stress-related genes. Functional analysis revealed enrichment in ER stress-related processes and pathways. Hub genes (BCL2, CCL2, IL-1β, TLR4, TNF, TP53) were identified, and immune infiltration analysis showed altered immune cell proportions. Validation in GSE60436 and *in vitro* confirmed ER stress gene dysregulation. Drug prediction suggested potential small molecules targeting ER stress markers.

**Conclusion:**

This study provides a comprehensive molecular characterization of ER stress in PDR, highlighting altered biological processes, immune changes, and potential therapeutic targets. The identified hub genes and small molecules offer avenues for further investigation and therapy development, enhancing understanding of PDR pathogenesis and aiding targeted intervention creation.

## 1 Introduction

Diabetic retinopathy (DR), a prevalent microvascular complication of diabetes mellitus (DM), contributes to visual impairment in approximately one-third of diabetic patients (1). It emerges as one of the most severe complications of diabetes, especially when advancing to Proliferative Diabetic Retinopathy (PDR) (2, 3). PDR is characterized by abnormal blood vessel growth in the retina, leading to the potential for vision loss and blindness (4). The intricate molecular mechanisms underlying the transition to PDR remain a subject of intense research interest. Understanding the gene expression patterns and immune landscape associated with PDR is essential for unraveling the complexities of its pathogenesis and identifying potential therapeutic targets.

The endoplasmic reticulum (ER) serves as a cellular organelle responsible for protein homeostasis, or “proteostasis” (5). Cellular stress and inflammation can result in the buildup of unfolded or misfolded proteins, a condition known as ER stress (6). One of the underlying molecular mechanisms contributing to the pathogenesis of PDR is ER stress (7). Despite the recognized importance of ER stress in PDR, a comprehensive molecular understanding of ER stress-related biomarkers in the context of PDR remains a significant research gap (8–10). In recent years, molecular investigations into the intricacies of ER stress-related biomarkers have provided a promising avenue for understanding the molecular basis of PDR (5, 11, 12). Unraveling the specific biomolecular signatures associated with ER stress in PDR holds the potential not only to deepen our comprehension of disease mechanisms but also to identify precise targets for therapeutic intervention.

Despite significant strides in diabetes research, there remains a gap in our understanding of the specific molecular events that drive the progression to PDR. Advancements in high-throughput technologies have revolutionized our ability to dissect the molecular landscape of complex diseases (13). Through the analysis of the transcriptome profiles of PDR patient samples and normal samples in the GSE102485 dataset from the GEO database, we investigated differentially expressed genes (DEGs) related to ER stress in PDR. Through Gene Ontology (GO) enrichment analysis, Kyoto Encyclopedia of Genes and Genomes (KEGG) pathway analysis, and Protein-Protein Interaction (PPI) network analysis, our objective was to enhance our understanding of the molecular characteristics of ER stress-related biomarkers in PDR.

Six key genes were identified through STRING, Cytoscape and CytoHubba, and further validation was performed in a separate dataset (GSE60436) and in a DR model using *in vitro* quantitative real-time polymerase chain reaction (qRT-PCR). Additionally, we explored the correlation between these central genes and the level of immune cell infiltration, revealing the immunomodulatory role of ER stress in PDR. Finally, potential small molecules for treating PDR were predicted using the Connectivity Map (cMAP). The objective of this analysis was to identify drugs with potential therapeutic effects that may intervene in the development of PDR by modulating molecular pathways associated with ER stress. This study bridged molecular biology and DR research, aiming to dissect the molecular signatures indicative of ER stress in PDR and shed light on the nuanced interplay between ER stress and the progression of DR.

## 2 Methods

### 2.1 Data collection

Two independent PDR datasets were downloaded from the GEO database. (http://www.ncbi.nlm.nih.gov/geo/), which included GSE102485 and GSE60436 (**Table 1**). The first transcriptome dataset was the test dataset (GSE102485) and the second microarray dataset was the validation dataset (GSE60436). For GSE102485, we selected a subset of 22 neovascular proliferative membrane specimens and three normal retina samples (14). We downloaded and analyzed the raw data from the GSE102485 dataset, processed and normalized the protein-coding genes using the R package “DESeq2” for further analysis. Additionally, we selected the GSE60436 microarray dataset as the validation set, comprising 6 PDR samples and 3 normal samples (15). The raw data from the GSE60436 dataset were downloaded, underwent ID conversion, normalization, and background correction. Additionally, we identified 328 ER stress-related genes with a relevance score greater than 3 in Genecards (**Supplementary Table S1**).

**Table 1:** Data information.

Data	Platform	PDR	Normal	Other
GSE102485	GPL18573	22	3	5
GSE60436	GPL6884	6	3	0

### 2.2 Identification of ER stress-related DEGs

Before conducting differential analysis on GSE102485, we filtered for mRNAs with expression counts greater than one in at least the number of replicates. Subsequently, we analyzed gene expression using the DESeq2 package. DEGs were identified using the criteria of an adjusted P-value <0.05 and an absolute |log2 Fold Change| ≥ 2. Next, we employ the R software packages “heatmap” and “ggplot2” to create visual representations, including heatmap and volcano plots. We performed an intersection analysis and created a Venn diagram to visually represent the overlap among these ER stress-related genes and DEGs in GSE102485.

### 2.3 Functional and pathway enrichment analysis

We conducted GO enrichment analysis and KEGG pathway analysis on the ER stress-related DEGs using the R package “ClusterProfiler” (16). We considered adjusted p-values of < 0.05 to be statistically significant in our analysis. Furthermore, we employed the Metascape database (https://metascape.org/) to further explore the functional mechanisms. The criteria set were a minimum overlap of 3, p ≤ 0.01, and a minimum enrichment of 1.5.

### 2.4 PPI Network construction and hub gene identification

We used the STRING database (https://string-db.org/) to explore the interactions among the ER stress-related DEGs (17). A threshold of a combined score ≥ 0.4 was set to identify significant interactions between these genes. The Cytoscape plugin MCODE was employed to filter out important modules of core genes in the PPI network with the following criteria: degree cutoff = 2, node score cutoff = 0.2, K-core = 2, maximum depth = 100. To identify hub genes, we utilized the CytoHubba plug-in (version 0.1) within Cytoscape software. CytoHubba identifies hub genes based on the consensus of multiple algorithms (MCC, MNC, Degree and EPC).

### 2.5 Analysis of immune cell infiltration

We utilized the CIBERSOFT algorithm from the R package “IOBR” (18) to analyze the infiltration of multiple immune cell subtypes in PDR and control samples from the GSE102485 dataset with the transcriptome of neovascular membranes. Linear regression analysis was conducted to analyze the correlation between the expression of ER stress-related hub genes and immune cells. The results were visualized using the R package “ggplot2”.

### 2.6 Cell culture

Human umbilical vein endothelial cells (HUVECs) (ATCC, Cat. CRL-1730) were cultured in DMEM containing 10% fetal bovine serum (FBS) and 1% antibiotic-antimycotic under standard conditions (5% CO2, 37°C). The HUVECs in the logarithmic growth phase were categorized into two groups for the experiment: the control group and the high glucose (HG) group. HUVECs cultured in a medium with 5.5 mmol/L glucose were assigned to the control group, whereas those cultured in a medium containing 30 mmol/L glucose were assigned to the HG group.

### 2.7 Western blot analysis

Protein extraction from HUVECs was performed using RIPA lysis buffer containing protease inhibitors and phosphatase inhibitors (Yesen, China). The protein concentration was determined using the BCA protein assay kit (ZJ102, Epizyme, China). Subsequently, proteins were separated on 10% SDS-PAGE gels and transferred onto PVDF membranes (R9A84148, Millipore). The membranes were then blocked in QuickBlock™ Western Blocking Buffer (P0252, Beyotime) for 30 minutes and incubated overnight at 4°C with primary antibodies against TRAM1 (18243-1-AP, Proteintech) and TXNIP (12705-1-AP, Proteintech). The following day, the membranes were incubated with HRP-conjugated secondary antibodies at room temperature for 1 hour. Protein bands were visualized using enhanced chemiluminescence (SQ201, EpiZyme, China), and protein densitometry was quantified using ImageJ software (version 6.0; Media Cybernetics, Inc.). β-actin (66031-1-lg, Proteintech) was used as the internal reference.

### 2.8 RNA extraction and qRT-PCR

RNA was extracted from HUVECs using the EZ-press-RNA purification kit (EZBioscience, USA) according to the manufacturer’s plan. Then, cDNA was reverse-transcribed from total RNA using the reverse transcription kit (Takara, Japan) and RT-PCR was conducted using TB Green^®^ Premix Ex Taq™ II kit (Takara, Japan). Primer sequences are detailed in **Supplementary Table S2**. β-actin was chosen as the reference gene for normalizing mRNA expression levels, and quantitative analysis was performed using the 2^-ΔΔCT^ method.

### 2.9 Small molecular drug analysis for ER stress-related DEGs

The Connectivity Map (cMAP) website was utilized to investigate small molecule drugs with the potential to inhibit the formation and progression of PDR (19). We submitted the 51 ER stress-related DEGs to the cMAP website, focusing specifically on the 46 upregulated genes, to identify potential small molecule drugs that could inhibit the formation and development of PDR. The score in the results list returned by cMAP represents the percentage by which the reference gene set is more similar to the current perturbation compared to the similarity of the query to the current perturbation. Drugs with negative scores and high absolute values are considered potential treatments because they can inhibit the expression of ER stress characteristic genes.

### 2.10 Statistical analysis

An independent Student’s t-test was employed to compare the two groups based on statistically significant differences of normally distributed variables. For non-normally distributed variables, the Wilcoxon rank-sum test was utilized. Statistical analysis was performed using R (version 4.2.0), with a significance threshold set at p < 0.05.

## 3 Results

### 3.1 Identification of DEGs and ER stress-related DEGs

**Figure 1:**
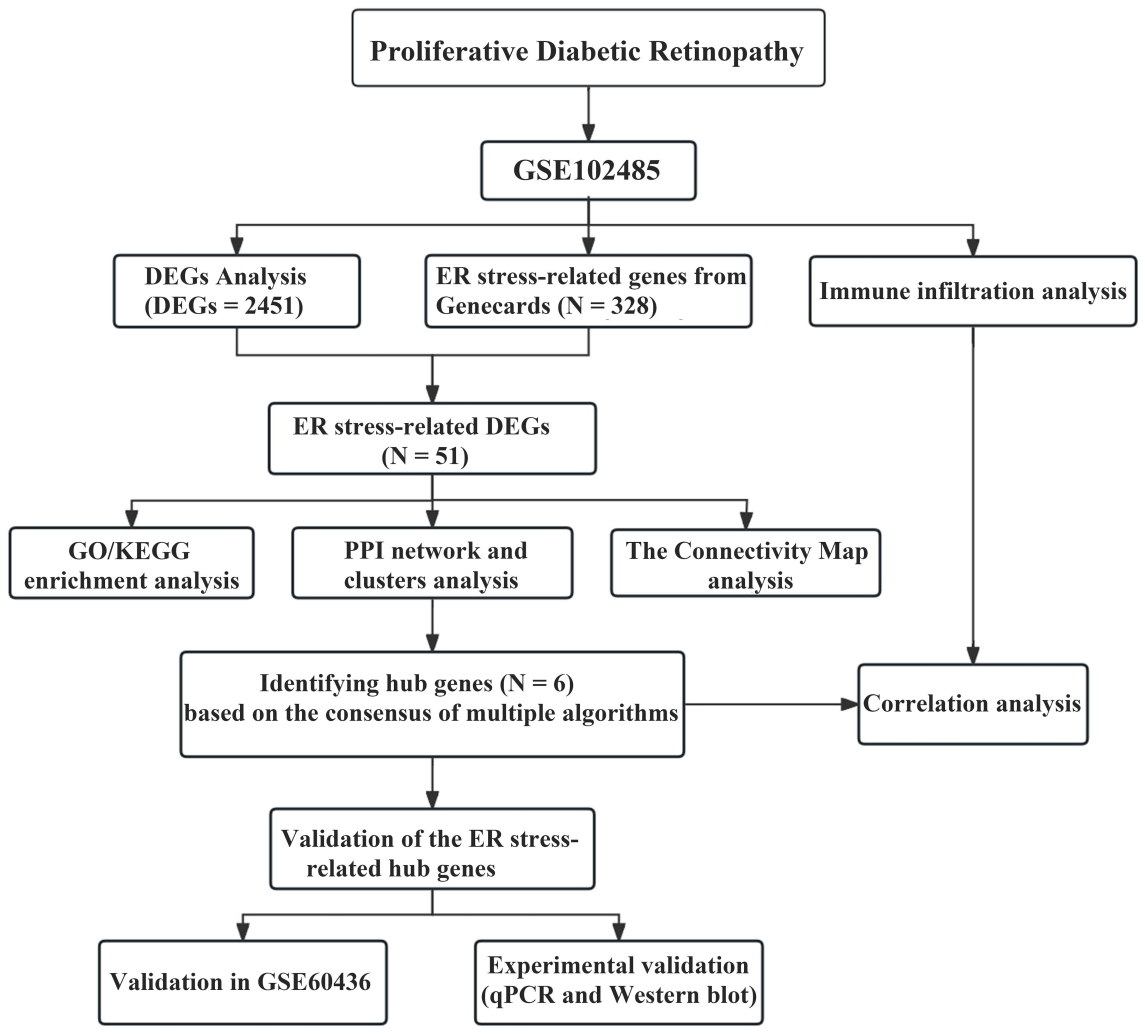
The workflow of our research.

**Figure 2:**
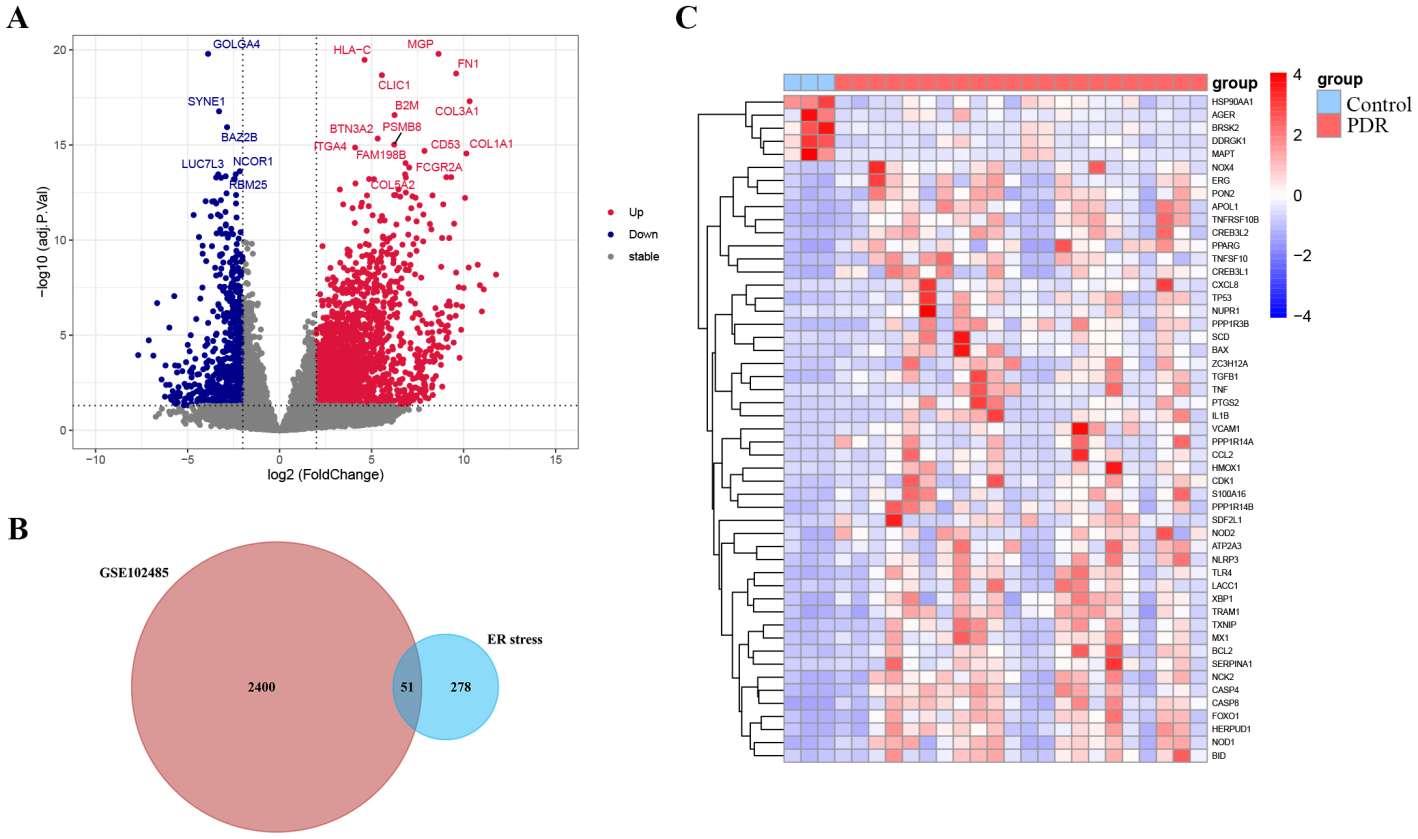
Identification of endoplasmic reticulum stress-related differentially expressed genes (ER stress-related DEGs). **(A)** Volcano plot of the DEGs in GSE102485. Blue dots represent downregulated DEGs, red dots represent upregulated DEGs and gray dots show genes with no significant difference. **(B)** Venn diagram of the intersection of DEGs in GSE102485 and ER stress-related genes. **(C)** Heatmap of the identified 51 ER stress-related DEGs in GSE102485.

The study was designed according to the flow chart outlined in **Figure 1**. Overall, 2451 DEGs were recognized in GSE102485, with 1815 genes displaying significant upregulation and 636 genes exhibiting significant downregulation. The volcano plots illustrating the DEGs are presented in **Figure 2A**. Subsequently, employing a filtering criterion of a correlation score > 3, we identified a total of 328 ER stress-related genes from the GeneCards database. By generating Venn diagrams, a total of 51 ER stress-related DEGs were identified, among which 46 were upregulated and 5 were downregulated as ER stress-related DEGs (**Figures 2B, C**).

### 3.2 Functional enrichment analysis of the ER stress-related DEGs

**Figure 3:**
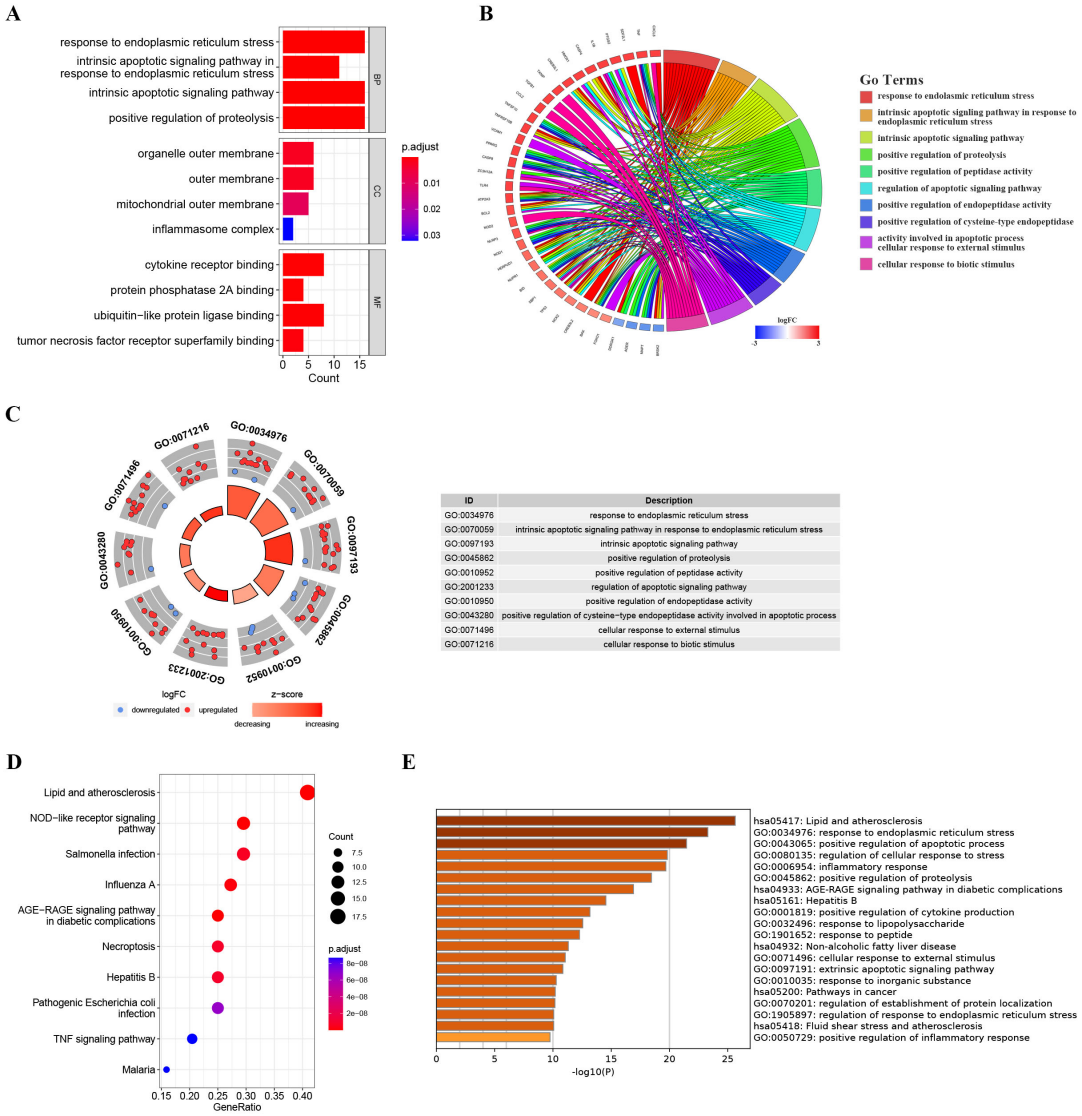
Enrichment analysis of ER stress-related DEGs. **(A)** Bar plot of enriched GO terms. **(B)** Chord diagram showing the relationships between enriched GO terms and associated genes, with colors indicating gene expression changes. **(C)** Chordal graph depicting expression changes of genes associated with GO terms; points’ colors reflect upregulation or downregulation. The table lists GO term IDs and descriptions. **(D)** KEGG analyses showing the enriched associated signaling pathways. **(E)** Metascape bar chart of the top 20 non-redundant enrichment clusters. The x-axis represents the -log10(p) value. The y-axis lists the GO terms and KEGG pathways associated with each cluster.

To further explore the potential biological functions of these ER stress-related DEGs at the biological level, we conducted GO and KEGG analyses. As depicted in **Figures 3A–C**, Biological Process (BP) terms were predominantly enriched in “response to endoplasmic reticulum stress”, “intrinsic apoptotic signaling pathway in response to endoplasmic reticulum stress”, and “intrinsic apoptotic signaling pathway”. In the context of Cellular Component (CC) ontology, significant enrichment was noted in both the “outer membrane” and the “mitochondrial outer membrane”. Turning to Molecular Function (MF) analysis, the predominant enrichment was identified in functions related to “cytokine receptor binding” and “protein phosphatase 2A binding”. Moreover, illustrated in **Figure 3D**, the KEGG analysis showcased enrichment in pathways such as the “AGE-RAGE signaling pathway in diabetic complications”, “NOD-like receptor signaling pathway” and the “TNF signaling pathway”. As illustrated in **Figure 3E**, the bar chart depicts the results of Metascape enrichment analysis for the provided gene list. The enrichment analysis of ER stress-related DEGs revealed significant associations with biological processes such as lipid and atherosclerosis pathways, immune responses including NOD-like receptor signaling and infectious diseases like influenza. Additionally, ER stress-related DEGs were implicated in cellular responses to various stimuli, including abiotic and mechanical stress, as well as pathways related to ER stress and apoptotic signaling.

### 3.3 Identification and analysis of ER stress-related hub genes

**Figure 4:**
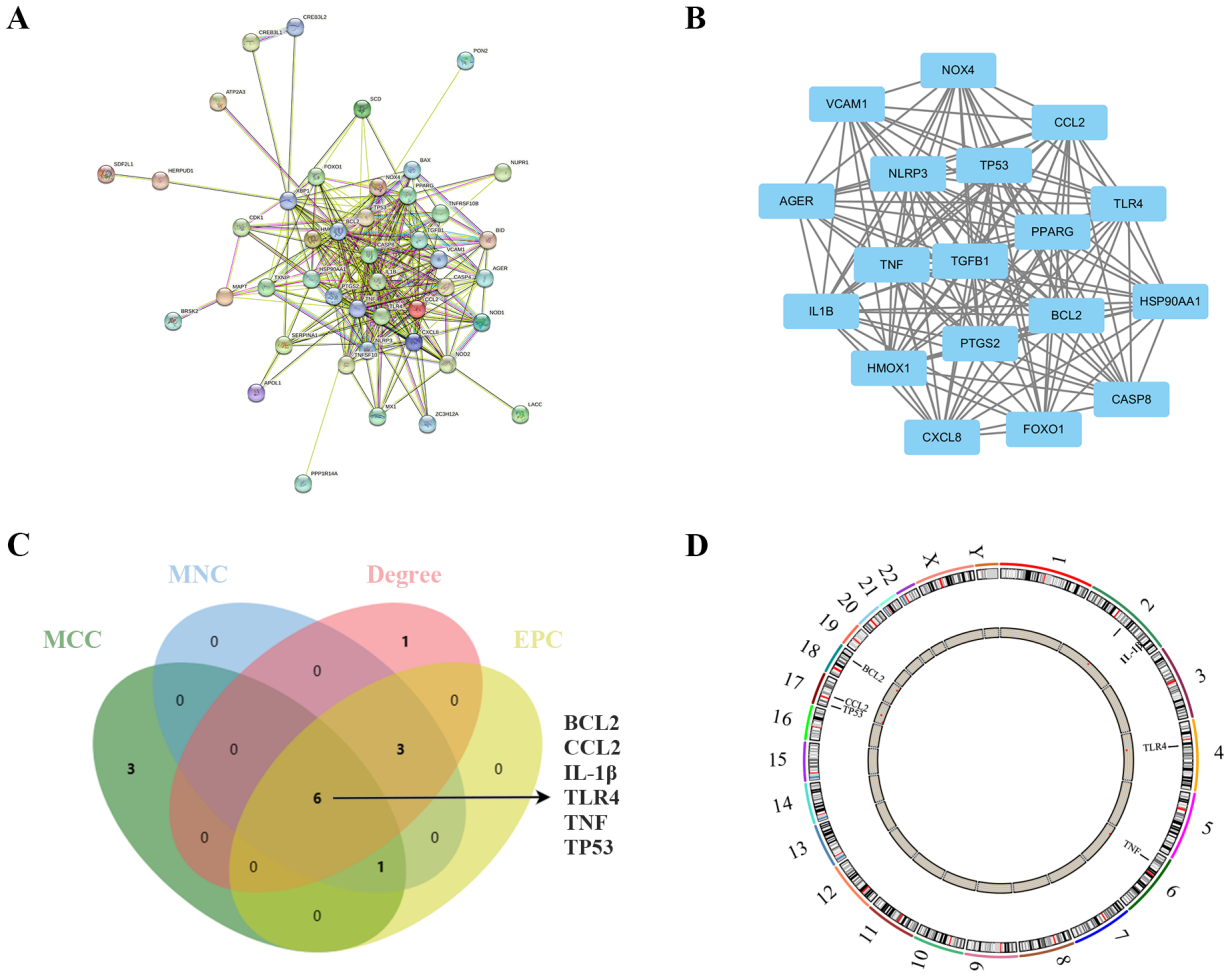
Identification and analysis of ER stress-related hub genes. **(A)** PPI network of ER stress-related DEGs. **(B)** Subnetwork of hub genes from the PPI network. **(C)** Identification of six candidates for hub genes by four algorithms. **(D)** The location of the 6 hub genes on the 22 chromosomes.

To further elucidate the potential relationships among the proteins encoded by these ER stress-related DEGs and to identify hub genes, a PPI network analysis was conducted using STRING. The PPI network comprised 51 nodes and 283 edges, with a highly significant enrichment (PPI enrichment p-value < 1.0e-16) as shown in **Figure 4A**. Subsequently, module analysis using the MCODE plugin revealed the most significant module: Module 1, which included 18 nodes and 140 edges, with a cluster score of 16.471 (**Figure 4B**).

In the quest to pinpoint the hub genes among the ER stress-related DEGs, multiple topological analysis algorithms, including MCC, MNC, Degree, and EPC, were employed. The results from the top 10 genes obtained from each algorithm were cross-referenced, leading to the identification of 6 hub genes: BCL2, CCL2, IL-1β, TLR4, TNF, and TP53 (**Figure 4C**). The location of the 6 hub genes on chromosomes is shown in **Figure 4D**.

### 3.4 Immune infiltration analysis

**Figure 5:**
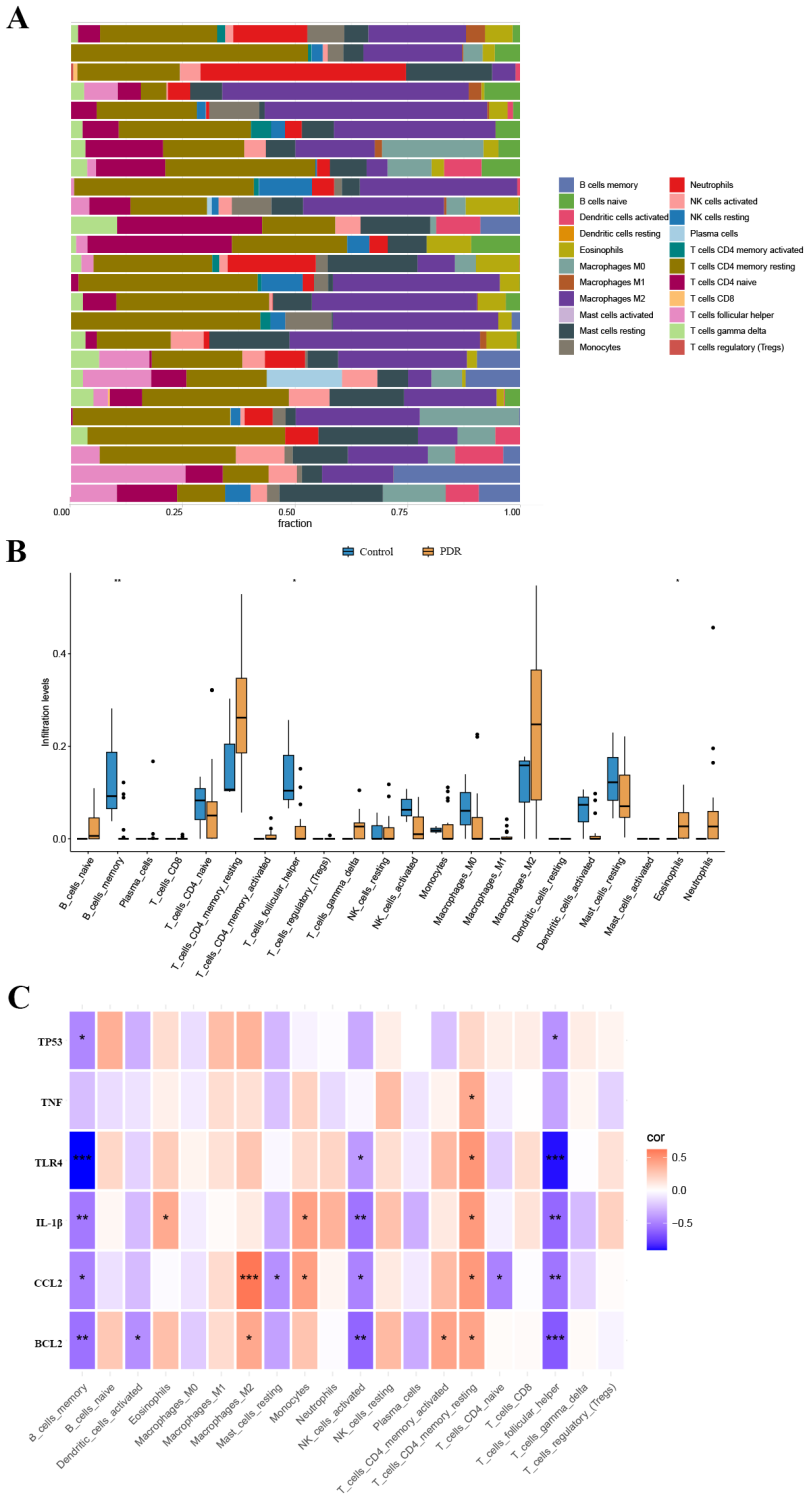
The landscape of immune cell infiltration. **(A)** The abundance of 22 immune cells in PDR samples and control samples. **(B)** The fraction of each immune cell type in the two groups. **(C)** Correlation between ER stress-related hub gene expression and immune cells. *p < 0.05; **p < 0.01; ***p < 0.001.

We used the CIBERSORT algorithm to assess the proportions of different infiltrating immune cell types between the PDR group and the control group. The bar chart presented in **Figure 5A** illustrates the proportions of 22 immune cell types across the 25 samples. Compared to the control group, there was an increase in eosinophil infiltration while memory B cells and T follicular helper cell infiltration decreased (**Figure 5B**). Concerning the correlation between hub gene expression and immune cell infiltration, there was a notable negative correlation between the expression of BCL2, CCL2, IL-1β, TLR4, and TP53, and the levels of infiltration of memory B cells and T follicular helper cells (**Figure 5C**).

### 3.5 External validation of TRAM1, TXNIP and ER stress-related hub genes

**Figure 6:**
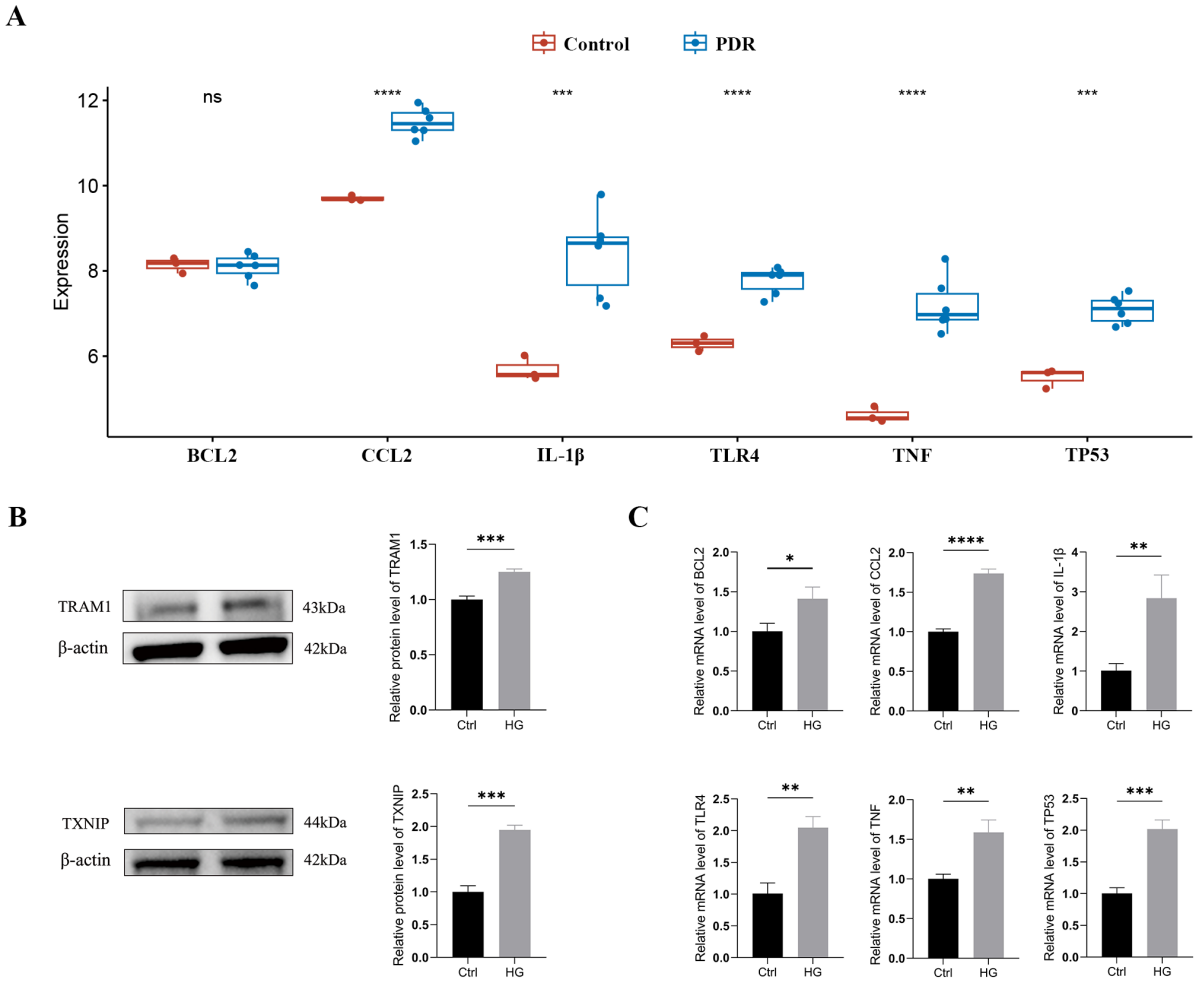
External validation of TRAM1, TXNIP and ER stress-related hub genes **(A)** Validation of ER stress-related hub genes in the GSE60436 dataset. **(B)** The protein levels of TRAM1 and TXNIP were evaluated in cell samples by western blot. **(C)** The mRNA levels of BCL2, CCL2, IL-1Β, TLR4, TNF, and TP53 were measured in cell samples by qRT-PCR. Ctrl, control group; HG, high-glucose group. *p < 0.05; **p < 0.01; ***p < 0.001; ****p < 0.0001.

Typically, there is a degree of consistency observed between the outcomes of the training set and those of the validation set. After normalizing the raw data from the validation set GSE60436, we found that in the comparison between the PDR group and the control group, the expression differences of CCL2, IL-1β, TLR4, TNF, and TP53 were consistent with our results from the GSE102485 dataset, and these differences were statistically significant with a p-value < 0.05 (**Figure 6A**).

In addition, we cultured HUVECs in a HG environment (30mM) to simulate the DR model *in vitro*. Firstly, we examined the expression of the ER stress markers TRAM1 and TXNIP proteins using Western blotting, which are key executive factors of ER stress. The results revealed a significant upregulation of TRAM1 and TXNIP protein levels in HUVECs after incubation with high glucose (**Figure 6B**), indicating the occurrence of ER stress. Subsequently, we employed qRT-PCR analysis to validate the expression of ER stress-related hub genes. The results revealed a significant upregulation in the expression of BCL2, CCL2, IL-1β, TLR4, TNF, and TP53 in HUVECs after 48 hours under HG conditions compared to the low-glucose environment (**Supplementary Table S3**). These findings align with the results obtained from bioinformatics analysis (**Figure 6C**).

### 3.6 Drug prediction for ER stress signature

**Figure 7:**
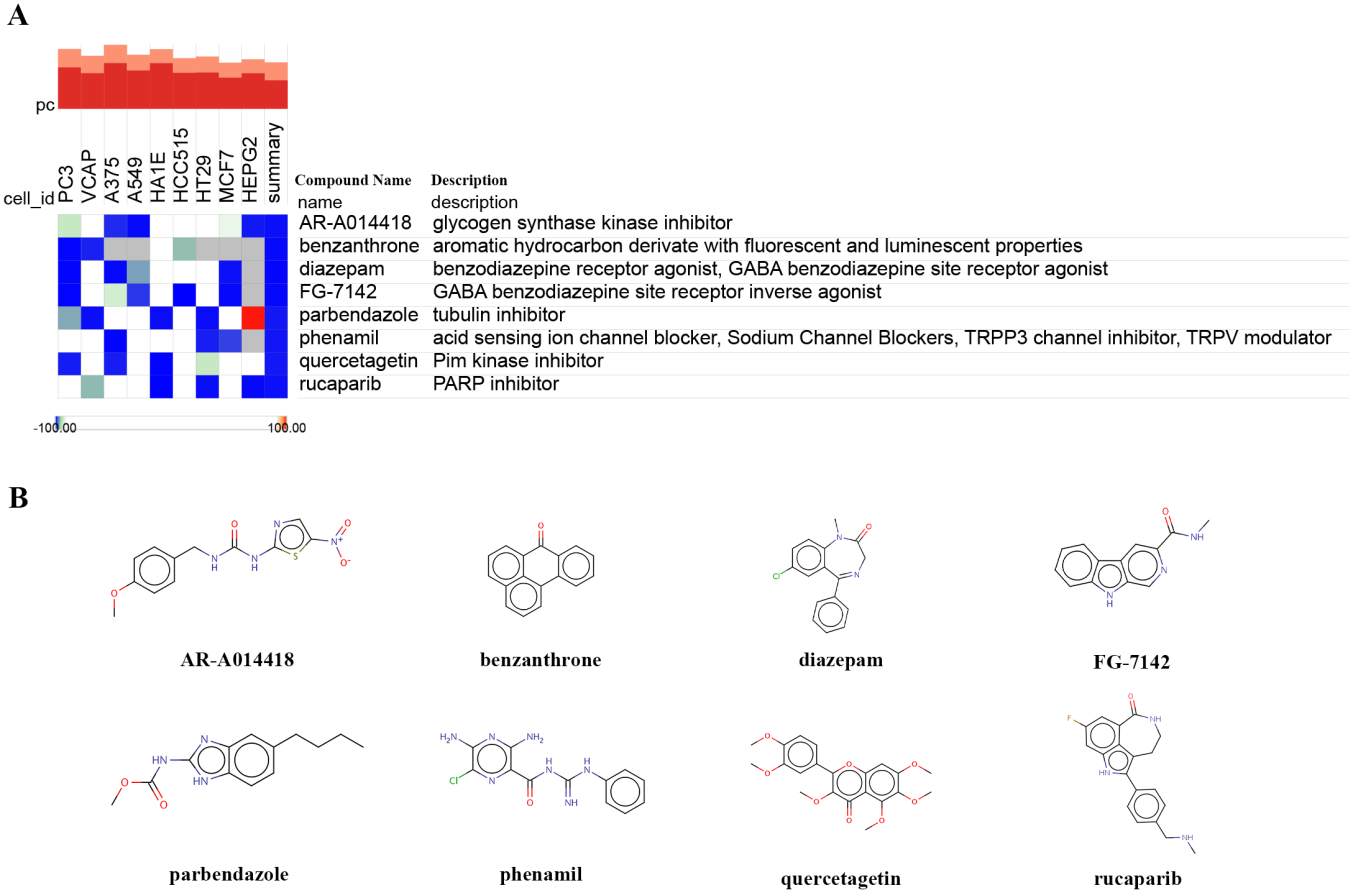
Identifying small-molecule compounds via cMAP analysis. **(A)** A heatmap illustrates the top 8 negatively enriched compounds. **(B)** The chemical structures of these 8 compounds.

To predict potential small molecule drugs that may inhibit ER stress in PDR, we uploaded the upregulated ER stress-DEGs to the cMAP online tool. We identified 8 drugs with the highest negative scores (diazepam, FG-7142, benzanthrone, AR-A014418, rucaparib, phenamil, quercetagetin, and parbendazole), indicating that they may inhibit the expression of ER stress markers (**Figure 7A**, **Supplementary Table S4**). Furthermore, we present the chemical structures of these eight small molecular compounds (**Figure 7B**).

## 4 Discussion

DR is a common microvascular complication in diabetic patients, characterized by abnormalities in retinal blood vessels (20). Although the exact pathological mechanisms of this disease are not fully understood, high blood glucose levels are considered its major triggering factor. The retina, as a highly metabolically active tissue, is sensitive to light and rich in polyunsaturated fatty acids, making it susceptible to oxidative stress (21). Emerging research underscores the critical role of ER stress in maintaining cellular homeostasis (22). Furthermore, excessive and prolonged ER stress is closely associated with the increased risk of various acute and chronic eye diseases, such as DR, cataracts, glaucoma, age-related macular degeneration, and others (23–25). Moreover, the involvement of ER stress in microenvironment regulation underscores its potential influence on additional ocular inflammatory conditions, such as uveitis and keratitis (5, 26). Recent studies have shown that dysregulated ER stress regulation has become one of the major contributors to the development of DR (9, 10). This understanding provides important clues and potential targets for the development of new therapeutic strategies.

Bioinformatics methods are increasingly utilized for aiding disease diagnosis and exploring potential therapeutic targets for PDR (27, 28). To the best of our knowledge, while biomarkers related to autophagy, cell pyroptosis, and ferroptosis have been investigated in DR, there is currently no reported bioinformatics analysis focusing on ER stress-related genes in DR (29–31). Our study delves into the intricate molecular landscape of PDR through a comprehensive analysis of gene expression profiles, ER stress-related DEGs, immune cell infiltration, and potential therapeutic targets. The amalgamation of multiple datasets, GSE102485 and GSE60436, empowered us to unravel the complex interplay of molecular events underlying PDR pathogenesis.

The identification of 328 ER stress-related genes, along with the subsequent focus on 51 DEGs, enhanced our understanding of ER stress in PDR. The upregulation of 46 genes associated with ER stress suggests a pivotal involvement of this cellular stress response in the disease, emphasizing its potential as a therapeutic target. The GO and KEGG pathway analyses illuminated the biological processes and pathways associated with ER stress-related DEGs. Enrichment in ER stress response, apoptotic signaling pathways, immune-related processes, and pathways related to lipid and atherosclerosis underscored the multifaceted nature of PDR pathogenesis. The Metascape enrichment analysis further expanded our comprehension, linking ER stress-related DEGs to broader biological processes and diseases. In fact, cholesterol levels are elevated in the blood of type 2 diabetes patients, and there was a significant increase in lipid peroxides in the vitreous humor of patients with PDR (32, 33). Due to the presence of various lipid-processing enzymes in the ER, the accumulation of free cholesterol and phospholipids rich in saturated fatty acids on the ER membrane occurs during lipid overload. This results in ER stress and increased mitochondrial β-oxidation, triggering the generation of reactive oxygen species (ROS), thus being closely associated with the severity of the disease (34, 35).

The construction of a PPI network and identification of hub genes shed light on the molecular interactions and central players in PDR. BCL2, CCL2, IL-1β, TLR4, TNF, and TP53 emerged as pivotal hub genes, implicating their involvement in modulating the complex network of molecular events associated with PDR. The negative correlation between hub gene expression and specific immune cell types suggests potential immunomodulatory roles for these genes. The pro-apoptotic and anti-apoptotic BCL-2 family members have been demonstrated to localize to the ER (36, 37). Prolonged ER stress may lead to the phosphorylation of IRE1 through TRAF2 and ASK1, activating the c-Jun N-terminal kinase (JNK) pathway (38, 39). This further results in the phosphorylation of the BCL2 protein, activating pro-apoptotic members of the BCL2 family (such as Bad, Bak, Bax and Bok), ultimately causing cell damage or even apoptosis (40). When the ER stress signaling pathway is activated, it leads to the secretion of immune-suppressive and metastasis-related cytokines, such as CCL2, by tumor cells, reshaping the tumor microenvironment for immune cell evasion. However, the STING inhibitor (C-176, H151) can alleviate the ER stress response and reduce the secretion level of CCL2 in tumor cells with high chromosomal instability (41). During ER stress, the activation of the NF-κB pathway promotes the upregulation of NLRP3 and its substrate, IL-1β, in the NLRP3 inflammasome (42). Simultaneously, the dissociation of IRE1α and PERK from BiP facilitates the localization of NLRP3, leading to both NLRP3 activation and apoptosis (43). Studies have revealed that ER stress can enhance the production and secretion of TNF-α and IL-1β (44). Additionally, TLR4 activation is known to induce inflammatory responses and lead to the release of these inflammatory cytokines. Consequently, ER stress and TLR4 may collaboratively promote the occurrence of inflammatory reactions by regulating the production of TNF-α and IL-1β, thereby playing a role in the development of inflammatory diseases. The upregulation of TP53 exacerbates the elevation of ROS levels and calcium ion release in tumor cells, inducing ER stress imbalance and promoting cell death in colorectal cancer cells (45).

The ER stress has a significant impact on the immune system, with an important association between ER stress and immune dysregulation through maintaining ER homeostasis and enhancing sensitivity to inflammatory stimuli (46). The CIBERSORT algorithm allowed for a comprehensive exploration of immune cell infiltration in PDR. Consistent with previous research results, changes in eosinophil infiltration and the negative correlation between hub gene expression and memory B cells and follicular helper T cells provide insights into the immune landscape associated with PDR (47, 48). These findings underscore the intricate crosstalk between ER stress and immune responses in the context of PDR pathogenesis.

A recent study suggests that TCF7L2 acts as a trigger factor for ATF6-related ER stress signaling. The upregulation of TCF7L2 expression influences the permeability of HUVECs by activating ATF6-related ER stress signaling (49). Furthermore, we further analyzed the upregulation of ER stress-related markers TRAM1 and TXNIP in HUVECs under high-glucose conditions, indicating that ER stress is one of the pathological mechanisms of DR. The validation of our findings in an independent dataset (GSE60436) and *in vitro* experiments on HUVECs underscored the robustness of our bioinformatics analysis. The consistent upregulation of hub genes in both datasets highlights their potential as reliable biomarkers for PDR. Furthermore, the identification of potential small molecule drugs, such as diazepam and rucaparib, offers promising avenues for therapeutic intervention in mitigating ER stress in PDR.

While this study provides valuable insights into the gene expression patterns and immune landscape associated with PDR, it is important to acknowledge certain limitations. Firstly, the relatively limited sample size may impact the generalizability of the findings. Additionally, the utilization of specific datasets and analytical tools in this study introduces the potential for dataset-specific biases, and alternative datasets or analysis methods might yield different outcomes. Future research endeavors should focus on expanding sample sizes, validating findings, employing more comprehensive study designs and analytical approaches to gain a more nuanced understanding of the molecular mechanisms and immune regulation in PDR.

## 5 Conclusion

In conclusion, our integrated analysis provides a holistic view of the molecular and immune landscape associated with PDR, with a particular focus on ER stress. The identified hub genes and potential therapeutic targets offer valuable insights for future research and the development of targeted interventions in the context of PDR. Further experimental validation and clinical investigations are warranted to translate these findings into tangible clinical applications.

## Data Availability

The original contributions presented in the study are included in the article/**Supplementary Material**. Further inquiries can be directed to the corresponding authors.
